# Long-term night shift work, genetic predisposition and risk of incident asthma: a prospective cohort study

**DOI:** 10.1093/qjmed/hcae098

**Published:** 2024-05-10

**Authors:** J Wang, Y Chen

**Affiliations:** The Second Clinical Medical College, Zhejiang Chinese Medical University, Hangzhou, China; The Second Affiliated Hospital of Zhejiang Chinese Medical University, Hangzhou, China

We read with great interest the article by Hu et al. entitled ‘Long-term night shift work, genetic predisposition and risk of incident asthma: a prospective cohort study’.^1^ The study revealed that current and lifetime night shift work may increase the risk of developing asthma, regardless of genetic predisposition. However, we have some concerns with this study and look forward to addressing them.

First, we know that both viral and bacterial infections can trigger or aggravate asthma.^2^ The study follow-up ended on 31 December 2021, however, with the outbreak and spread of COVID-19 in 2019, more than one-third of COVID-19 patients developed persistent respiratory symptoms after being infected with SARS-CoV-2.And in a national population cohort study in South Korea, COVID-19 was associated with a higher incidence of new-onset asthma.^3^ The study did not adequately assess the prevalence of basal respiratory infections in the study population. However, other potential confounders associated with the development of asthma, such as allergies, pregnancy and psychological evaluation, which were not addressed in this study, could have influenced the findings.^4^

Second, some patients with atypical asthma symptoms, manifested by cough or chest tightness, are easily missed in diagnosis and statistics, which can also be problematic. The large number of participants in the study made the study more convincing, but the fact that most were from the UK affected the generality of the findings.

Finally, we appreciate the remarkable work by Hu et al. However, we would like to alert readers to the potential limitations of this study and sincerely hope to get their response.

## References

[1] Hu B , Xie Y, Yin H, Yang S, You X, Ma J, et al Long-term night shift work, genetic predisposition, and risk of incident asthma: a prospective cohort study. QJM 2024; 117:631–637. 10.1093/qjmed/hcae068 38597880

[2] Singh SJ , Baldwin MM, Daynes E, Evans RA, Greening NJ, Jenkins RG, et al Respiratory sequelae of COVID-19: pulmonary and extrapulmonary origins, and approaches to clinical care and rehabilitation. Lancet Respir Med 2023; 11:709–725. 37216955 10.1016/S2213-2600(23)00159-5 PMC10198676

[3] Kim BG , Lee H, Yeom SW, Jeong CY, Park DW, Park TS, et al Increased risk of New-Onset asthma after COVID-19: a nationwide Population-Based cohort study. J Allergy Clin Immunol Pract 2024; 12:120–32.e5. 37774780 10.1016/j.jaip.2023.09.015

[4] Almeida ML , Santana PA, Guimarães AM, Gurgel RQ, Vianna EO. Asthma and pregnancy: repercussions for neonates. J Bras Pneumol 2010; 36:293–300. 20625665 10.1590/s1806-37132010000300005

